# Chia Chi Wang and his research on Protozoology

**DOI:** 10.1007/s13238-020-00759-x

**Published:** 2020-07-10

**Authors:** Ting Shi, Lei Fu

**Affiliations:** Zhejiang Normal University, Jinhua, 321004 China

**Figure 1:**
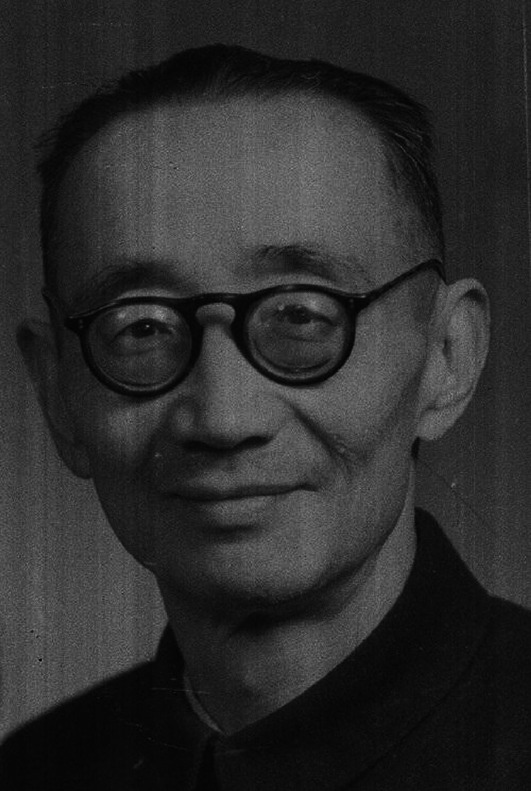
**Chia Chi Wang (1898–1976)**

Chia Chi Wang (王家楫, 1898–1976) was a famous protozoologist, the founder of protozoology and pioneer of rotiferology in China (Fig. 1).

Chia Chi Wang was born in a scholarly family in Fengxian, Jiangsu. At the age of six, he began to study at the Zhaowen Academy. When the country was declining, his father advocated that scholars should learn advanced technology to change the status quo. His father’s pioneering spirit and enlightened attitude towards new things had a profound impact on Chia Chi Wang’s life.

In 1920, Chia Chi Wang graduated from the Nanking Ecole Normal School (which was later incorporated into Southeast University), where he later engaged in protozoology-related research under the guidance of Professor Chih Ping (秉志). In 1925, he studied at the University of Pennsylvania as a state-financed student because of his excellent performance. While in the United States, he delved into the study of marine protozoa. In 1928, his doctoral thesis *Ecological Studies of the Seasonal Distribution of Protozoa in a Freshwater Pond* (Wang, 1928) won the Gold Medal, and earned him a doctorate. While studying in the United States, he published a number of papers on the physiology and taxonomy of protozoa in *Science*, *Morphology and physiology*, as well as *Animal Physiology*, which attracted much attention from American scholars. Later, he was hired as a Sterling Fellow at Yale University.

In 1929, Chia Chi Wang heard that foreign scientific groups would go to China to collect plant and animal samples. He thought that Chinese biological resources belonged to China, and claimed foreigners should know that Chinese affairs could be solved by Chinese people, and that biological resources in China could be exploited by Chinese people only. Therefore, he gave up the superior job opportunities and living conditions provided by Yale University, and returned to China to continue his research on protozoa. Then, Chia Chi Wang was hired as a professor of zoology at the Institute of Biology of the Science Society of China, and a professor of biology at the National Central University at the same time. In 1934, he served as a director of the Institute of Zoology and Botany of the National Academia Sinica. Later, an advanced research program on the fish, protozoa and algae of China was initiated at this institute, which was headed by Chia Chi Wang. In 1948, he was elected as an academician of the National Academia Sinica. In 1949, he was appointed as a standing committee member of the National Academia Sinica and the director of the Institute of Zoology. In the same year, the Institute of Hydrobiology of the Chinese Academy of Sciences was established and Chia Chi Wang was appointed as the director. In 1955, he was elected as an academician of the Chinese Academy of Sciences.

Chia Chi Wang was a great biologist, who was the first to study protozoa in China, and the founder of rotiferology. Under the influence of Chih Ping, Chia Chi Wang developed a great interest in Darwin’s theory of evolution. He devoted himself to this theory, and personally went into the field to study protozoa. When he found that the research on protozoa in China was still underdeveloped, he decided to dedicate to this field. In 1925, Chia Chi Wang’s first paper *Study of the Protozoa of Nanking* (Wang, 1925) was published, and this was the first article on protozoology in China, which meant the beginning of Chinese protozoology research. In Pennsylvania, his PhD thesis *Ecological Studies of the Seasonal Distribution of Protozoa in a Fresh-water Ponds* was a continuation of his research on protozoa, which he later continued after returning to China. In the following four years, he travelled around Shandong, Fujian, Guangdong, Sichuan and other coastal areas and territorial seas of China to study protozoa. As a result, many marine and freshwater protozoa were discovered, which laid the foundation for future studies. Later, he found that ciliophora still retain a fiber membrane although the cilia are degenerated. Subsequently, he published the paper *the fiber system of the wall worm*. His research played an important role in accelerating the development of protozoan phylogeny. Chia Chi Wang suffered from severe cataract and 1,200-degree myopia in his later years, but he never stopped his research, and then addressed *Protozoa in the Everest Region* (Jiaji, 1974) and *Protozoa in parts of the Tibetan Plateau* (Jiaji, 1977), which made great contributions to the investigation of protozoa in Tibet. Moreover, he discovered three new genera, fifty-eight new species, four new varieties, and eight new subspecies of protozoa, illustrating the extent of his groundbreaking work.

**Figure 2:**
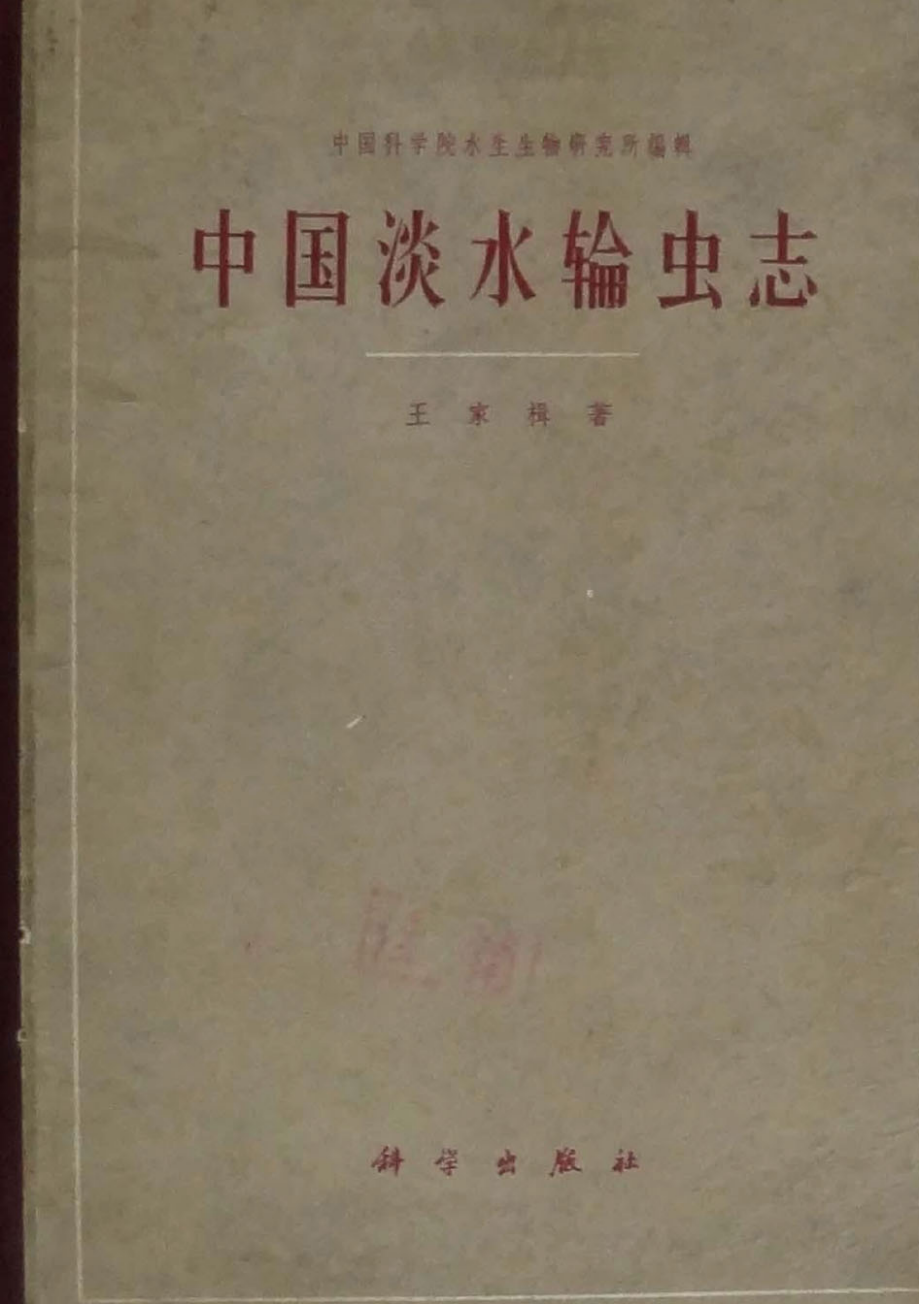
**Chia Chi Wang’s book** ***Chinese Freshwater Rotifers***

Chia Chi Wang was the pioneer of Chinese freshwater rotiferology. His book *Chinese Freshwater Rotifers* (Jiaji, 1961) (Fig. 2), which was the first to classify and describe the common rotifer species in Chinese swamps, ponds, lakes and reservoirs in detail, expanded the area of Chinese invertebrate zoology. This book included 252 species divided into 79 genera and 15 families. There were 4 new species and 2 new races, which were summed up in detail from class to genus with a search index attached. The description of the species was particularly detailed. One or more images were attached to all of the 252 species, with 533 pictures in total, which were classified into 27 plates. The kinship, morphology, physiology and ecology of rotifers was discussed in depth, and this work won him the National Science Conference Award in 1978, as well as the Hubei Science Conference Award.

**Figure 3:**
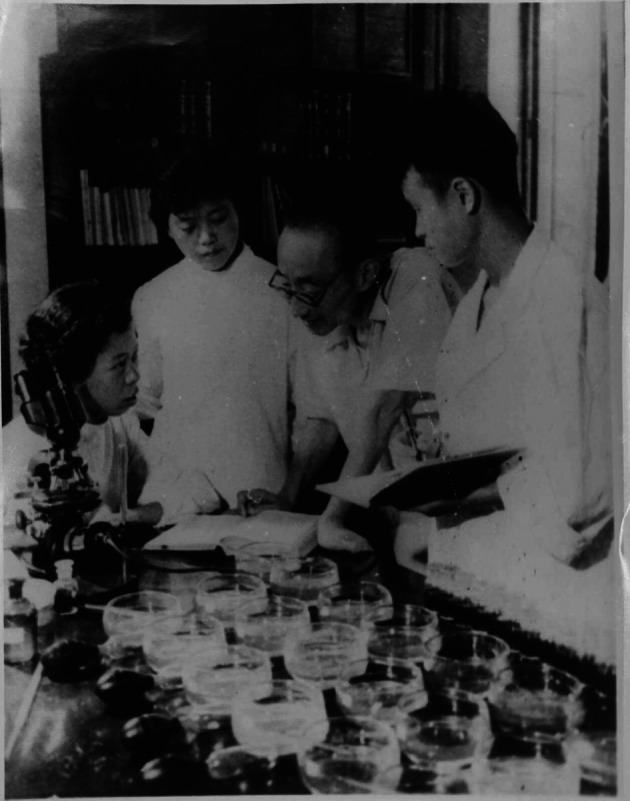
**Chia Chi Wang and his students**

Chia Chi Wang’s contribution was not limited to his own research, since he also instructed several famous biologists (Fig. 3), including Zhu Shuping (朱树屏) and Shen Yunfen (沈韫芬). In 1927, Chia Chi Wang taught general biology, invertebrate zoology, histology and embryology at the Institute of Biology of the Science Society of China and the National Central University. Under the influence of his father, he believed that only teaching knowledge through textbooks rather than in person didn’t have any effect. Thus, he emphasized that the goal of university education was not only imparting textbook knowledge, but also life skills, to improve the students’ ability to solve the urgent problems of the nation (Wang, 1934, 1936), which was especially important at the birth of modern China.

Accordingly, Chia Chi Wang was also an influential social activist. In 1934, the China Zoological Society was founded, and he served as a board council member as well as the editor-in-chief of *the Journal of Zoology*. In 1952, he became a member of the Jiu San Society (九三学社). In 1954, he was the team leader of the group in Wuhan, and the chairman of the Wuhan Branch. From December 1960 to February 1961, he helped set up the Central Fisheries Research Institute for the Democratic Republic of Vietnam. In 1962, he participated in the second meeting of the Western Pacific Fisheries Commission in Leningrad, Soviet Union.

Chia Chi Wang is clearly one of the great biologists of China, an educator and a patriotic scholar. He was a role model for Chinese zoologists. He made an indelible historical contribution to Chinese protozoology and rotiferology.
